# Developing treatments for cerebral small vessel disease: a scoping review of licensed interventions for potential repurposing

**DOI:** 10.12688/f1000research.157890.2

**Published:** 2025-09-10

**Authors:** Philip M Bath, Elizabeth P Phan, Gwynneth Clay, Jesse Dawson, Paresh Malhotra, Rob Howard, Suvankar Pal, Joanna M Wardlaw, Terry Quinn, Malcolm Macleod

**Affiliations:** 1 Stroke Trials Unit, University of Nottingham, Nottingham, England, NG7 2UH, UK; 2 Stroke, Nottingham University Hospitals NHS Trust, Nottingham, England, NG7 2UH, UK; 3 School of Cardiovascular and Metabolic Health, University of Glasgow, Glasgow, G4 0SF, UK; 4 School of Cardiovascular and Metabolic Health, University of Glasgow, Glasgow, G51 4TF, UK; 5 Division of Neurology, Imperial College, London, W6 8RP, UK; 6 Maple House, University College London, London, England, W1T 7NF, UK; 7 Anne Rowling Regenerative Neurology Clinic, University of Edinburgh, Edinburgh, Midlothian, EH16 4SB, UK; 8 Dementia Research Institute Centre, University of Edinburgh, Edinburgh, Midlothian, EH16 4SB, UK; 9 CAMARADES Group, University of Edinburgh, Edinburgh, Midlothian, EH16 4SB, UK

**Keywords:** cerebral small vessel disease; isosorbide mononitrate, metformin, tadalafil, treatment, repurposing

## Abstract

**Background:**

Cerebral small vessel disease (cSVD) is a progressive neurovascular-degenerative condition without specific treatment that causes lacunar stroke, most intracerebral haemorrhage, vascular cognitive impairment (VCI) and several neuropsychiatric conditions.

**Objectives:**

To conduct a rapid multi-stage scoping review to identify licensed interventions that could be repurposed for testing in cSVD at phase-3.

**Methods:**

First, we screened preclinical studies of potential relevance to cSVD and used a drug dictionary to identify studies of potential interventions. Separately, we screened clinical studies of relevance to cSVD and VCI. Following merging, we removed drugs that were unsuitable or impractical to assess long-term in the UK. We then performed mini-meta-analyses for shortlisted interventions assessing effects on cognition and scored these for their relevance to cSVD.

**Results:**

The preclinical review created a long-list of 1,757 deduplicated interventions. Those that were available in the UK, not expensive or impractical to administer long-term were merged with 62 interventions identified from 75 relevant clinical studies to create a medium-list of 52 interventions. Focussed literature review short-listed ten interventions for review by an Independent Scientific Advisory Group; they ranked three as most suitable for immediate testing: metformin, tadalafil and isosorbide mononitrate.

**Conclusion:**

This rapid review identified three interventions that are suitable for testing in a late phase-3 (platform) trial involving patients with cSVD. The approach could be improved with partial automation, text mining and generative pre-trained transformer approaches which would help manage the large data volumes. Further, our data-driven approach could be combined with genetic or other mechanistic methods to further de-risk future trials.

## Introduction

### Rationale

Dementia is the commonest known brain disorder with rates increasing as populations age: ^1^ world prevalence will treble by 2050 ^2^ and reach 1.7 million people in England & Wales by 2040. ^3^ Common types comprise Alzheimer’s disease (AD), vascular dementia (VaD) and mixed dementia, mostly combined AD and VaD. ^1^ Most vascular cognitive impairment (VCI) and VaD, with or without AD, is caused by cerebral small vessel disease (cSVD), ^4^ a progressive neurovascular-degenerative condition that is typically diagnosed on neuroimaging. ^5, 6^ cSVD increases the future risk of stroke, dementia and functional decline 2-to-3 fold. ^7^ cSVD, including that which is covert, is extremely common and estimated to affect 10% of the world’s population, i.e. approximately 750M worldwide, more so in low-middle income countries. ^8^ cSVD contributes to 45% of dementias. ^9^ Further, cSVD causes lacunar ischaemic stroke (25% of the 17M strokes/year worldwide), ^10^ most intracerebral haemorrhages (ICH, 10% of strokes) and underlies many mobility, gait, falls, neurobehavioral, mood/depression and urinary incontinence disorders in older people. ^1, 4, 9^ Importantly, the biggest concern expressed by patients with cSVD is the development of VCI/VaD. ^11^

Unfortunately, cSVD has no specific proven preventative or restorative interventions and lowering blood pressure only has a very limited effect. ^12– 14^ The only phase-3 trial was stopped early because dual antiplatelet therapy with aspirin and clopidogrel caused an increase in deaths in patients with prior lacunar ischaemic stroke. ^15^ This trial also found that blood pressure lowering did not reduce recurrent stroke ^16^ or modify cognition. ^17^ Recently, a phase-2 feasibility trial after lacunar stroke found that isosorbide mononitrate (ISMN), especially when given with cilostazol, improved cognition and functional outcome and reduced recurrent stroke. ^18^

Given the long, expensive path to new drug development, there is increasing focus on repurposing existing drugs. Repurposing may be “defined as researching new indications for already approved drugs or advancing previously studied but unapproved drugs” ^19^ and offers the advantages of assessing interventions which already have a wealth of preclinical and clinical data for another indication. Repurposing drugs can be faster, less expensive and risky and carry higher success rates than traditional drug development approaches primarily because researchers can bypass earlier stages of development that establish drug safety. ^20^ As a result, repurposing reduces research waste.

Hence, an increasingly common approach to identifying interventions for testing in neurodegenerative conditions such as cSVD, Alzheimer’s disease, multiple sclerosis and motor neuron disease are structured systematic approaches that identify candidate treatments on the basis of their known mechanisms of action and existing pre-clinical and clinical studies. ^12, 21– 24^

### Objectives

To update and expand on our previous mechanistic assessment ^12^ with a wider scoping review of medications that might be repurposed as effective treatments for cSVD with a focus on cognitive outcomes.

## Methods

### Background

We conducted a rapid multi-stage scoping review ^25^ to identify potential interventions for preventing cognitive decline in people with cSVD with the aim of testing these in a phase-3 platform trial. Funding calls for platform trials typically specify key criteria to consider when selecting interventions for testing. We based our review on the criteria set out by the UK NIHR Health Technology Assessment programme for “large, collaborative and ambitious platform studies in areas of strategic importance” (number 23/95, call date 27 July 2023, closing date for outline applications 28 November 2023). ^26^ Key requirements were: i) “a clear process and Independent Scientific Advisory Group (ISAG) for the identification and prioritisation of candidate interventions”; ii) “evaluation of the clinical (and cost) effectiveness of multiple interventions” (implying that interventions should not be expensive); and iii) “each technology [ *intervention*] must have sufficient clinical data”, ^26^ i.e. that there will be a reasonable chance that it would be effective. Further, interventions should have already been tested in a NHS setting; be phase-3 ready; be available to be implemented rapidly if the trial is positive; be immediately useful to service users; treatment length should be commensurate with a platform design; it would be likely that the interventions would be used if supported by the results; and that there should be collective learning, hence the importance of sharing our process of identification and prioritisation of interventions for cSVD, as here. The following processes were designed to identify suitable phase-3-ready interventions that might modify cognition and could be tested in a platform trial and so fit the above call and its requirements.

### Registration

Since this was performed as a rapid scoping review to be completed within two months, we did not create a prior protocol. Additionally, as a scoping review, we did not register it with PROSPERO.

### Overview

**Figure 1.**
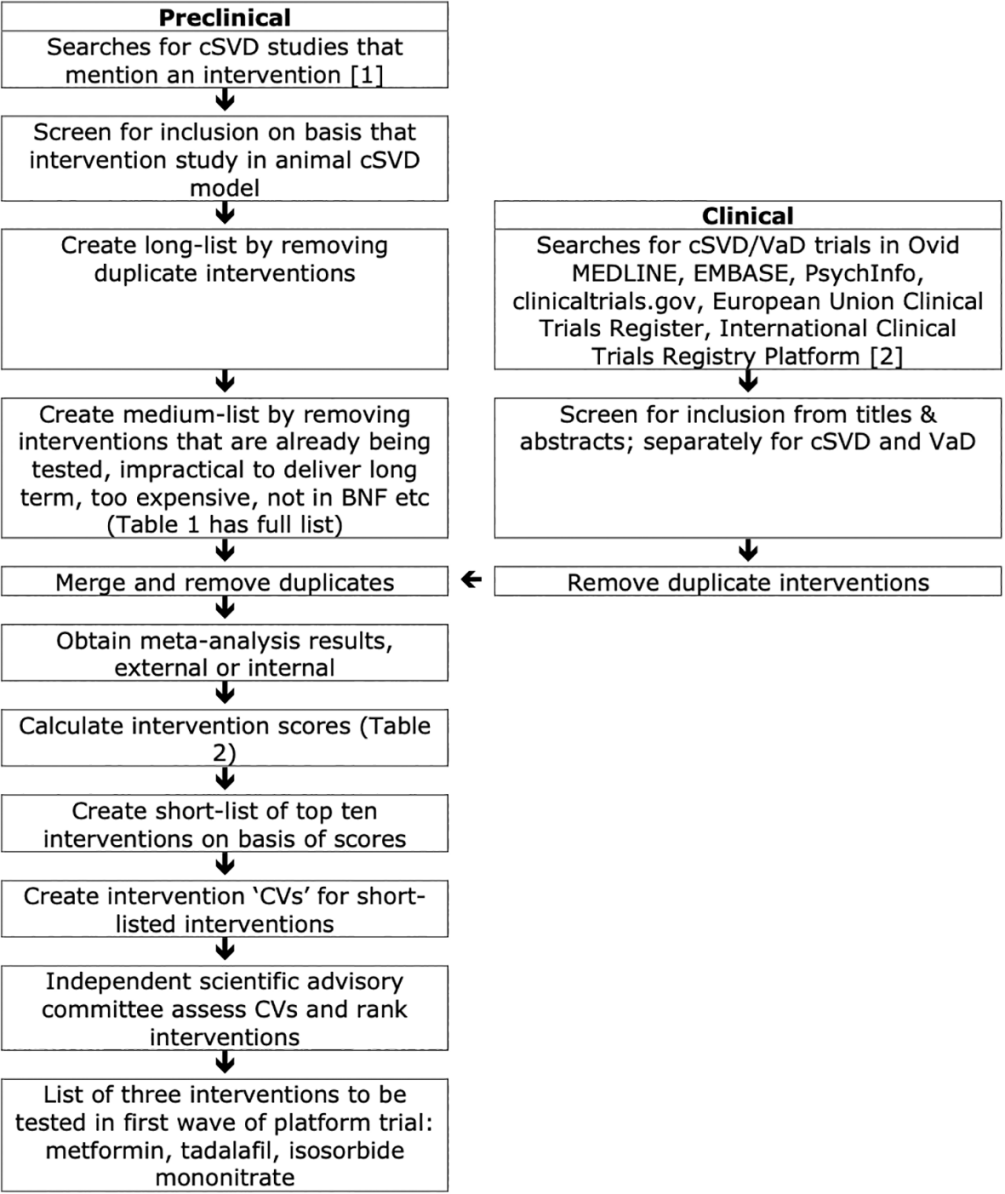
Structured identification of potential interventions for treatment of cerebral small vessel disease.

The intervention selection strategy is summarised in Figure 1. Interventions of potential relevance to cSVD from preclinical and clinical searches were merged to form a long-list. From this, interventions were excluded for several reasons including that they were not available in the UK or could not be delivered feasibly. The resulting medium-list was then scored and ten interventions with the highest scores were assessed by an ISAG who identified three that should be taken forward into a potential multi-arm multi-stage platform trial. Searches had no date limits but were primarily limited to English due to the time constraint.

### Preclinical workpackage

Building on our previous work, ^27^ we (MM) searched PubMed on 14/08/23 (search terms in Figure 1), and loaded Titles and Abstracts to R, before text-mining using a series of regular expressions representing over 12000 compounds derived from DrugBank ^28^ and from the Alzforum Therapeutic Database ( https://www.alzforum.org/therapeutics) ( Figure 1, top left). Using a similar approach, we text-mined titles and abstracts using regular expressions to identify publications using rats, mice or primates, to create a list of interventions tested in preclinical models. Our focus was primarily on medications; lifestyle, dietary manipulation and cognitive rehabilitation were not considered in scope.

To generate the medium-list, interventions were removed if they would be impossible or impractical to deliver in a phase-3 platform trial based in the UK or another similar socialised healthcare system. A full list of reasons for removal is given in Table 1 and includes drugs that were not listed in the British National Formulary (BNF ^29^) in quarter 3 2023, were considered too expensive for widespread use (threshold BNF price of £30 per month) and interventions that were considered impractical to deliver long-term.

**Table 1.** Reasons for excluding interventions from the long-list identified from preclinical searches.

Reason	Justification	Example(s)
Agents in same class	Opportunity cost to co-develop agents in same class	Isosorbide mononitrate vs isosorbide dinitrate
Not in BNF ^29^	Not available in UK so not available to the trial and could not be implemented rapidly if trial positive	AChE-i: phenserine. CCB: nilvadipine. PDE4-i: ibudilast. ROCK-i: Fasudil
Impractical to administer	Impractical to administer to large populations	Electromagnetic stimulation. Hyperbaric oxygen. Remote ischaemic conditioning
Intravenous administration	Impractical to administer to large populations	Alpha-galactosidase. Antipsychotics. Glucagon. Prostacyclin
Side effect profile	Significant or severe side effect profile where benefit might not outweigh risk	Anti-cancer. Anti-convulsant: oxcarbazepine. Pyridone immune-suppressive: perfenidone
In widespread use: prescribed	Challenging to find patients who are not on the intervention, both for eligibility and avoidance in control group	ARA: candesartan, losartan, telmisartan. CCB: amiloride, nifedipine, nimodipine. Statins: atorvastatin
In widespread use: over-the-counter or via internet	Challenging to find patients who are not on the intervention, both for eligibility and avoidance in control group	Fatty acids: omega-3 fatty acids, poly-unsaturated. ^13^ PDE3-i: sildenafil
No evidence to chase	No evidence to suggest that might work in cSVD	Nootrope: piracetam. PCSK9-synthesis blocker: evolocumab. Xanthine oxidase inhibitor: allopurinol
Neutral in another neurological condition	Less likely to be effective in cSVD if neutral in another neuro-degenerative condition	ARA: losartan neutral for AD. ^47^ Benzothiazoles: riluzole neutral for MS. ^48^ CCB: nilvadipine neutral for AD. ^46^ Potassium-sparing diuretic: amiloride neutral for MS. ^48^ SSRI: fluoxetine neutral for MS ^48^
Planned or current testing in cSVD	Funder unlikely to support more testing	Anti-platelet: cilostazol
For short-term use	Long-term safety not confirmed	Anti-herpes: valaciclovir; valganciclovir
Not identified in preclinical search	No preclinical data to support clinical use	Anti-herpes: valaciclovir; valganciclovir
Too expensive for widespread use	Unaffordable to NHS for use in large populations	GLP-1: Liraglutide, semaglutide
Poor adherence	Reduced chance of success if intervention is not taken/used	GLP-1: Liraglutide, semaglutide

### Clinical workpackage

In parallel, we (EPPM, TQ) screened clinical trials assessing interventions separately for cSVD and VaD ( Figure 1, top right), again with the prime aim to improve cognition. The searches for cSVD and Vad trials were conducted between October 2021 - January 2022 and June - August 2022, respectively, and updated for both in June 2023. Completed trials were identified from Ovid MEDLINE, EMBASE and PsychInfo (for VaD only) databases for articles in any language from 2012 to the start of the search. Planned and ongoing trials were searched on clinicaltrials.gov, the European Union Clinical Trials Register (EUCTR) and International Clinical Trials Registry Platform (ICTRP) ( Figure 1). From these, we merged the identified interventions into two lists, one relevant to cSVD and the other for VaD. Interventions were classified by likely mechanism of action. Interventions were then merged with the preclinical medium-list.

### Mini-meta-analysis workpackage

The aim was to assess whether there was any evidence that individual interventions improved cognition or reduced cognitive decline in any medical indication. Published systematic reviews/meta-analyses of the effect of interventions on cognition were sought (PMB) irrespective of the outcome scale. If none were present, a rapid internal meta-analysis was performed using completed published data from randomised controlled trials or before-after studies identified from searches of the Cochrane Library and PubMed using controlled vocabulary and validated filters to identify RCTs. Data published as graphs rather than in tables were extracted on screen using mean and standard deviation (calculated from standard error if necessary) with screen values scaled for the Y-axis.

Data were entered into Cochrane Review Manager (version 5.4.1 for Mac) with the aim of identifying interventions that improved cognition, i.e. meta-analysis odds ratio for a good outcome (OR >>1 suggests benefit). For before-after studies assessing the effect of an intervention within a group of patients, the group size was split equally between the before and after results, a conservative approach. Binary data were analysed directly as an odds ratio (95% CI) using the Mantel-Haenszel method with fixed effects (i.e. assuming that the relative treatment effect was common across all included studies ^30^). Continuous data were analysed as standardised mean difference (SMD) with fixed effects to allow studies using different cognition scales to be integrated. Where necessary, scales were inverted to be compatible with direction of positive cognition, i.e. to reverse the direction of effect for scales that go from high to low. SMD and 95% confidence intervals (95% CI) were converted to an odds ratio (OR, 95% CI) so results from binary and ordinal/continuous results could be compared: ^31, 32^

OR=SMD×∏/√3=SMD×1.814

The sample size calculation for the planned UK STEP cSVD platform of N=1,460 was designed to detect an odds ratio of 1.4 (a ‘small’ treatment effect of relevance to a large population of cSVD) with 90% power; hence, short-listed interventions would need meta-analysis evidence that this could be achieved, i.e. OR≥1.4.

### Scoring of interventions workpackage

We developed (PMB) a bespoke scoring system to assess the likelihood that an intervention might successfully alter cognition in people with cSVD ( Table 2). The system comprised four parts: mechanisms, meta-analysis, identification of intervention for testing in another phase-3 platform, and feasibility. The first section comprised eight mechanistic targets in cSVD that interventions might modulate in an appropriate direction (e.g. pro-endothelial, anti-inflammatory) to potentially reduce the development and progression of cSVD pathophysiology ( Table 2), as used previously ^12^ (no additional mechanisms having been identified in the intervening nine years); the sectional-score ranges from 0-8. The second section comprised the results of mini rapid meta-analysis based on the odds ratio and lower limit of the 95% confidence interval, the latter to provide information on the precision of the meta-analysis ( Table 2); practically, the sectional-score based on the odds ratio ranges from above 0 to less than 100 with an odds ratio of >1 compatible with potential benefit. The third section reviewed whether any of three existing neurodegenerative platforms in the UK ACORD collaboration (MS-SMART, ^23^ MND-SMART ^24^ and OCTOPUS ^33, 34^) were testing the intervention or had plans to do so; the sectional-score ranges from 0-3. The reason for including this information was that it meant that an independent assessment had already identified the same class or intervention (even if this was based on over-lapping information sources). The scores for these three sections were then added to give a sub-score ranging from just above 0 to less than 100.

**Table 2.** Scoring system for assessing whether interventions might work in cSVD.

Attribute	Score	Notes
***Potential beneficial mechanisms***		Empirical scoring ^12^
Tighten blood brain barrier, BBB	+1	
Pro-endothelial	+1	
Antiplatelet	+1	
Smooth muscle cell, proliferative to contractile	+1	
Anti-leucocyte	+1	
Anti-fibroblast	+1	
Anti-inflammation	+1	
Anti-mitogenic	+1	
***Metaanalysis***		
Odds ratio, OR	OR	OR for a good outcome: OR >1 means potential benefit
OR 95% lower boundary, LB	OR 95% LB	OR 95% LB: LB >1 means significant benefit
***Identified for other platforms***		
MS SMART ^23^	+1	Two separate searching schemes identify same intervention
MND SMART ^24^	+1	Two separate searching schemes identify same intervention
OCTOPUS ^33, 34^	+1	Two separate searching schemes identify same intervention
***Sub-score 1 – sum of attributes***		Range 0 to <~100
***Feasibility***		
In British National Formulary (BNF) ^29^	x1	Essential for a phase-3 trial investigating repurposing
Acceptable side effects (from BNF ^29^)	x1	Judgement call, essential
Limited contraindications (from BNF ^29^)	x1	Judgement call, essential
Limited existing indication	x1	A widely used intervention would not be testable - essential
Can be delivered feasibly and long term	x1	Essential
Cost < £30 per month	x1	Judgement call for affordability for widespread use - essential
Not already being tested in cSVD	x1	Essential
***Sub-score 2 – multiplier of attributes***		Either 0 (impossible/impractical) or 1 (feasible)
***Total score = Sub-score 1 × sub-score 2***		Range 0 (infeasible) to <~100

The fourth section identified feasibility (yes = 1, no = 0) and required each and every attribute to be feasible including drug availability in the UK (for whatever indication), acceptable side effects, limited indications (<50% of population) and contraindications, the potential for long-term administration, reasonable treatment cost and not already being tested for cSVD ( Table 2). These were multiplied together to create a second sub-score with values or 0 (not feasible) or 1 (feasible). The two sub-scores were then multiplied to give the total score ranging from zero upwards. By example, if anything was considered non-feasible the total score would be zero whatever the results of the first three sections. Otherwise, if everything is feasible, then the final score will be driven by the sum of the first three sectional scores.

### Intervention CVs/profile workpackage

We created ‘CVs’ or profiles (PMB) for each shortlisted intervention comprising information on mechanisms of effect of potential relevance to cSVD (as previously ^12^), evidence of any clinical effect in cSVD, up to three recognised indications (or licensed indications for a drug) and meta-analysis findings (as above). Information from the BNF, ^29^ or equivalent source if a non-drug, was presented on: dosing; route of administration; NHS cost; adverse event profile; important drug interactions; and cautions, exclusions and contraindications. Information on adherence, score (as above) and any other relevant trials testing it was then given. Finally, a summary of pros and cons resulting from the CV was given.

### Independent review

To compare and rank the shortlisted interventions, an ISAG was created, this comprising experts in stroke or cognition/dementia and trials/platforms; membership comprised RH (chair), JD, PM and SP (academic medics) and GC (patient-public involvement). ‘CVs’ were distributed to the ISAG who then ranked them following open discussion at a video call; PMB was present in a non-voting role to clarify any information. The aim of ISAG was to consider drug summaries for up to 10 drugs, these comprising information of relevance to cSVD on: effects on target cells (e.g. endothelial), existing licensed indications, mini-meta-analysis results of trials in non-cSVD, feasibility of administration, pharmacology (including drug interactions, exclusions), testing in other platforms and overall score ( Table 5).

## Results

### Preclinical workpackage

**Figure 2.**
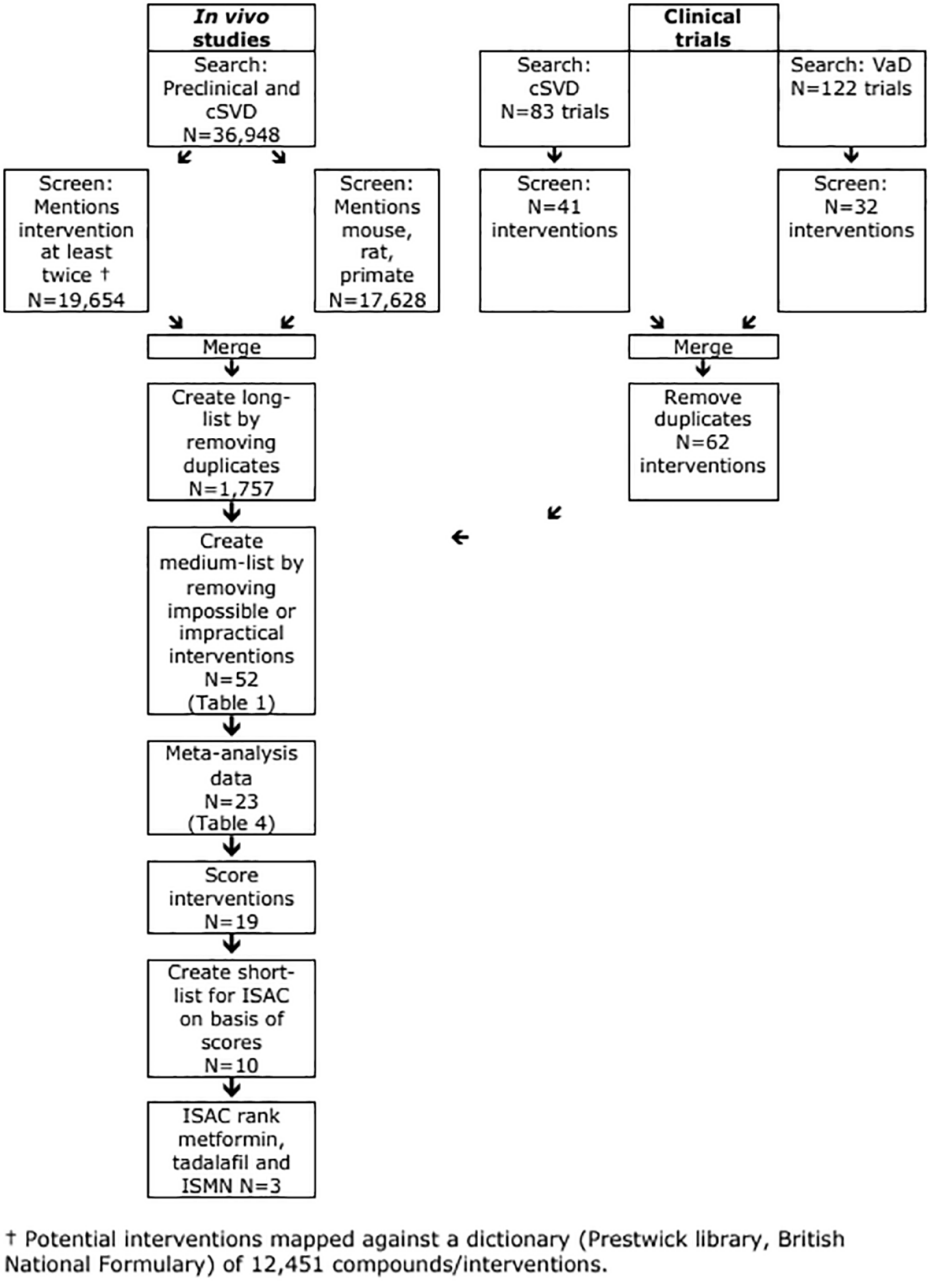
Flow of data showing number of interventions.

A search for preclinical references to cSVD (date 14 ^th^ August 2023) revealed 36,948 which reduced to 19,654 when limited to those with at least 2 mentions of an intervention included in our drug dictionary ( Figure 2, top left). 17,628 of 36,948 references included mention of rats, mice or primates, and of these 10,258 had mentions of 1,757 drugs included in our drug dictionary.

### Clinical workpackage

Searches of registries identified 82 randomised trials of relevance to cSVD with activity status - completed 28, completed with no results 0, suspended/terminated 3, ongoing 3, recruiting 27, to start 13, unknown status 8) ( Figure 2, top right). Identified interventions included six antihypertensive drugs, two antiplatelets (when given together), five cognitive enhancers/anti-dementia drugs, three involving electromagnetic stimulation, four physiological interventions, nine vasoactive drugs and 13 others of mixed types ( Table 3).

**Table 3.** Interventions identified in clinical searches of completed or ongoing trials for cSVD or VaD.

	For cSVD	For VaD
Antihypertensives	• Amlodipine • Atenolol • Azelnidipine + perindopril • Losartan • Telmisartan	• Amlodipine • Nimodipine • Valsartan
Antithrombotics	• Aspirin + clopidogrel ^15^	
Cognitive enhancers/anti-dementia	• Anisodine hydrobromide • Choline alphoscerate • Ginkgo - diterpene lactone or ketoester • Rivastigmine	• Almitrine-raubasine (Duxil) • Choline alphoscerate • Citicoline • Donepezil ^†^ • Galantamine • Ginkgo biloba • Memantine • Oxiracetam • Rivastigmine • Tandospirone citrate
Electro-magnetic stimulation	• Repetitive transcranial magnetic stimulation • Theta burst stimulation • Transcranial direct current stimulation	• Theta burst stimulation • Transcranial direct current stimulation
Physiological	• Hyperbaric oxygen treatment • Permissive hypercapnia • Pro-kin visual feedback balance training • Remote ischaemic conditioning (RIC) ^†^	• Remote ischaemic conditioning (RIC) ^†^
Vasoactive	• Alprostadil • Butylphthalide • Cilostazol ^18^ • Isosorbide mononitrate (ISMN) ^18 †^ • Pentoxifylline • Sarpogrelate hydrochloride • Sildenafil • Tadalafil ^†^ • Udenafil	• Angiotensin 1-7 • Butylphthalide • Nicergoline • Tadalafil ^†^ • Udenafil 41
Others	• Allopurinol • Atorvastatin • Carbogen • Equol • Exenatide • Heparin • Maraviroc • Minocycline ^†^ • Mixed tocotrienols • Mouse nerve growth factor • Phenylephrine • Polyethylene glycol loxenatide • Rosuvastatin ^†^	• Dapagliflozin • Equol (soy-based oestrogen containing supplement) • Ferrous succinate • Fluoxetine • Maraviroc • Methylphenidate • N-acetylcysteine • Olfactory ensheathing cells /Schwann cells/Olfactory receptor neurons • Prospekta (neurotransmitter modulator) • Rosuvastatin ^†^ • Urothilin A

^†^ Six of the interventions (or within the same class of interventions) shortlisted for consideration by the ISAG were present in both pre-clinical and clinical searches. However, lithium, metformin, semaglutide and vitamins B6/9/12 were shortlisted but not identified in cSVD/Vad clinical studies.

A parallel search for interventions of relevance to VaD identified 120 randomised trials with activity status - completed 38, completed with no results 0, suspended or terminated 1, ongoing 3, recruiting 50, to start 20, unknown status 8) ( Figure 2, top right). Identified interventions included three antihypertensive drugs, no antiplatelets, ten cognitive enhancers/anti-dementia drugs, two involving electromagnetic stimulation, one physiological intervention, five vasoactive drugs and 11 others of mixed types ( Table 3).

### Exclusion of interventions from long-list

Interventions from the preclinical and clinical searches were merged and de-duplicated to create a long-list. The long-list of potential interventions for cSVD was reduced to a medium-list by removing interventions that were impossible or impractical to study in a phase-3 platform trial running in the UK. Reasons and their explanations are given in Table 1 with the most common including drugs that are not listed in the British National Formulary and so not available to the trial (62 classes of intervention), drugs that need parenteral administration which would be impractical for widespread use (22 classes), interventions with a significant side-effect profile (15 classes), interventions without good existing preclinical or clinical evidence that the intervention might work (15 classes) and existing widespread use of the drug or class of drugs (13 classes).

### Mini-meta-analysis workpackage

The results of the mini-meta-analyses based on randomised controlled trials and before-after studies for the shortlisted interventions are shown in Table 4. Interventions varied in their potential to improve cognition for any indication; interventions with a large SMD/high OR were phosphodiesterase5-inhibitors whilst there was no evidence of efficacy for vitamin B12 (see Scoring spreadsheet, Data availability, CC-BY 4.0).

**Table 4.** Summary of meta-analyses for shortlisted candidate interventions for cSVD.

	Outcome	S	N	SMD (95% CI)	OR (95% CI) [ 1, 2]
AChE-i, any ^41^	Continuous	8	3796	0.23 (0.17, 0.30)	1.52 (1.36-1.72)
Donepezil 05mg		3	1556	0.20 (0.10, 0.31)	1.44 (1.20-1.76)
Donepezil 10mg		2	576	0.37 (0.19, 0.55)	1.96 (1.41-2.71)
Galantamine		2	966	0.26 (0.13, 0.39)	1.60 (1.27-2.03)
Rivastigmine		1	698	0.15 (0.01, 0.30)	1.31 (1.02-1.72)
Biguanide, metformin ( Figure 3a)	Continuous	6	650	0.44 (0.28, 0.58)	2.22 (1.66-2.92)
GLP-1, any	Binary	3	15820	-	2.04 (1.11-3.70)
Liraglutide		1	9340	-	2.00 (1.00-4.00)
Semaglutide		2	6480	-	2.13 (0.60-7.69)
GSK3-i, lithium	Continuous	5	289	0.51 (0.27, 0.75)	2.52 (1.63-3.90)
NMDA antagonist, memantine	Continuous	2	826	0.37 (0.23, 0.50)	1.96 (1.52-2.48)
NOD, isosorbide mononitrate ^18^	Ordinal	1	308	-	1.82 (1.16-2.78)
NOD + PDE3-i: ISMN + cilostazol ^18^	Ordinal	1	156	-	2.27 (1.18-4.35)
PDE5-i, any ( Figure 3b)	Continuous	4	110	1.14 (0.73, 1.55)	7.91 (3.76-16.63)
Tadalafil		2	34	1.21 (0.45, 1.96)	8.98 (2.26-34.99)
Udenafil [ 3]		2	76	1.11 (0.62, 1.60)	7.49 (3.08-18.21)
RIC, per/post-conditioning	Continuous	9	396	0.56 (0.36, 0.77)	2.76 (1.92-4.04)
Statin	Continuous	6	1599	0.20 (0.10, 0.30)	1.44 (1.20, 1.72)
Atorvastatin		5	923	0.20 (0.07, 0.33)	1.44 (1.14-1.82)
Rosuvastatin		1	676	0.20 (0.05, 0.35)	1.44 (1.10, 1.89)
Tetracycline, minocycline	Continuous	7	547	0.28 (0.11, 0.46)	1.66 (1.22-2.30)
Vitamin B12, post stroke	Continuous	2	6064	-0.03 (-0.08, 0.02)	0.95 (0.87-1.04)

AChE-i: acetylcholinesterase inhibitor; AD: Alzheimer’s disease; CI: confidence intervals; GLP-1: glucagon-like peptide-1 receptor agonist; GSK3-i: glycogen synthase kinase 3-inhibitor; ISMN: isosorbide mononitrate; N: number of participants; NOD: nitric oxide donor; OR: odds ratio; PDE-i: phosphodiesterase-inhibitor; PDE3-i: phosphodiesterase3-inhibitor; PDE5-i: phosphodiesterase5-inhibitor; RIC: remote ischaemic conditioning; S: number of studies; SMD: standardised mean difference; VaD: vascular dementia.

^1^ Odds ratio (OR) calculated from standardised mean difference: OR = SMD x ∏/√3. ^31, 32^

^2^ For drug classes with more than one constituent and sufficient data, only the drug with most data was assessed by the ISAG, e.g. donepezil was assessed but galantamine and rivastigmine were not.

^3^ Not available in UK.

**Figure 3.**
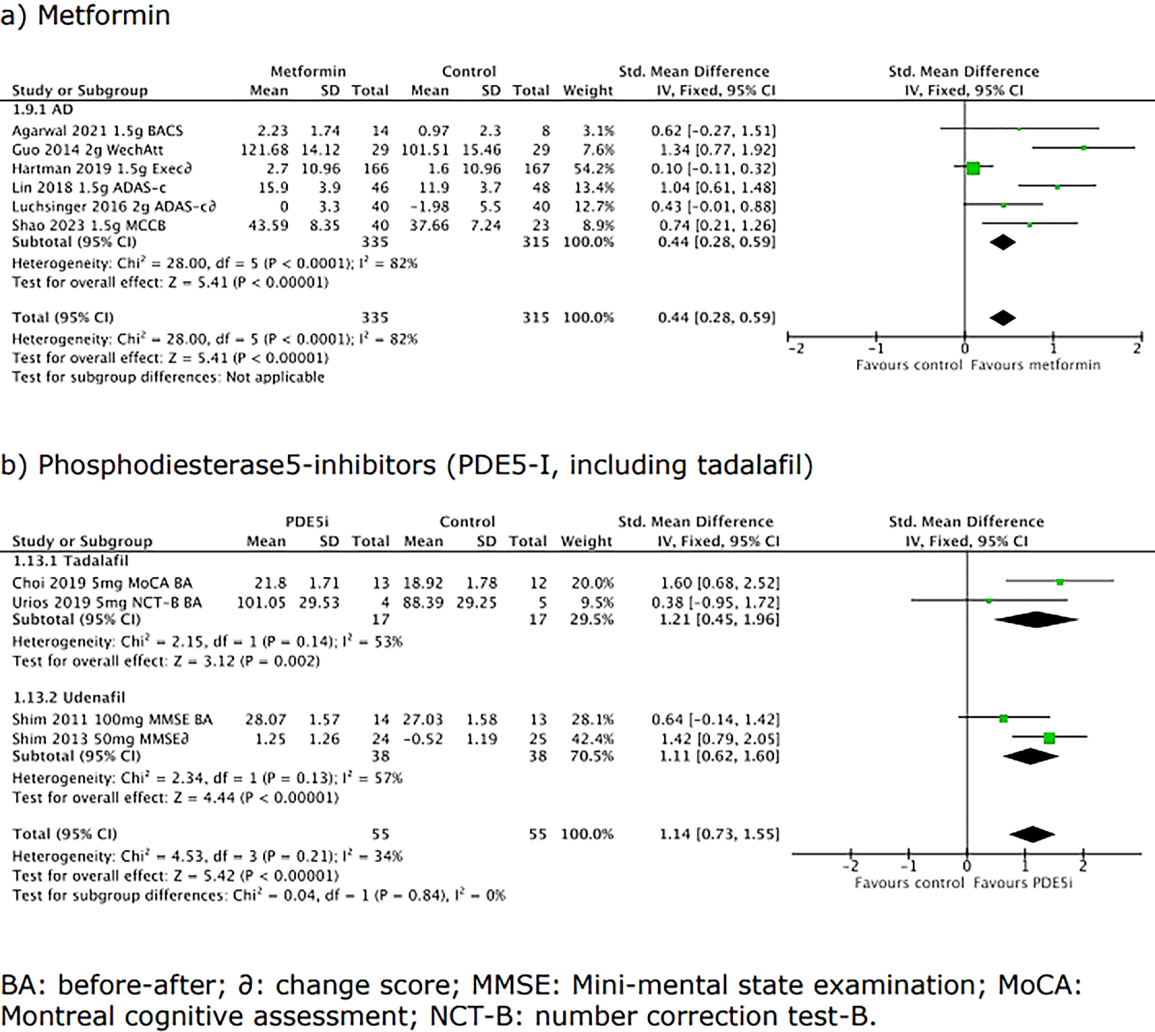
Forest plots for a) metformin, and b) phosphodiesterase-inhibitors (including tadalafil). Positive scores imply better cognition.

### Intervention CVs/profile workpackage

Summaries or “CVs” for 10 interventions were developed, as précised in Table 5.

**Table 5.** Characteristics of 10 shortlisted drugs as reviewed by Independent Scientific Advisory Group (ISAG). Characteristics shown here are a precis of the drug CV reviewed by ISAG.

Intervention	Cognition meta-analysis, cost, score and ranking	For testing	Against testing
***Ranked***			
Metformin (biguanide). No alternatives.	OR 2.22 (95% CI 1.66-2.92) n=650. Cost £2.17 pm. Score 21.5. Ranked 1.	Licensed first therapy in type 2 diabetes and for prevention of diabetes in pre-diabetes (500-1000 mg SR bd). Reduced cognitive impairment and cSVD burden in type 2 diabetes. ^59^ Epidemiology: reduced dementia in T2DM. ^60, 61^ Positive Mendelian randomisation study for AD. ^54^ Adherence 90.9% at 6+ months. ^62^ Being tested in OCTOPUS platform. ^33, 34^	Excludes diabetics on metformin. High dose (1g bd) associated with gastrointestinal side effects.
Tadalafil (phospho-diesterase5-inhibitor, PDE5-i). Alternatives: avanafil, sildenafil, vardenafil.	OR 7.91 (95% CI 3.76-16.63), N=110. Cost £1.93 pm. Score 47.1. Ranked 2.	Licensed for erectile dysfunction (5 mg prn), pulmonary hypertension (40 mg od), benign prostatic hypertrophy (5mg od). Adherence 88.7% at 24 weeks. Compatible with positive isosorbide finding in LACI-2 trial. ^18^ Meta-analysis positive on cognition in experimental AD. ^63^ Small single-dose studies show improvement in cerebral perfusion and blood flow. ^64, 65^ Medication-wide association study compatible with reduced dementia. ^56^	OR and hence score are very high - likely to be an over-estimate bearing in mind low sample size and 3 of 4 studies were before-after in design.
Isosorbide mononitrate (ISMN, nitric oxide donor, NOD). Alternatives: isosorbide dinitrate, glyceryl trinitrate.	OR 1.82 (95% CI 1.16-2.78), N=308. ^18^ Cost £ 6.75 pm. Score 18.7. Ranked 3.	Licensed for prophylaxis of angina and as an adjunct in congestive heart failure. Positive after lacunar stroke subtype of cSVD (LACI-2). ^18^ Adherence 86.2% at 12 months. Effect enhanced when given with cilostazol. ^18^ So could use combined ISMN & cilostazol.	Evidence for cognition in cSVD based on only one phase-2 trial, LACI-2. ^18^ Post ISAG meeting notes: 1. Since this study, LACI-3 has been funded by NIHR HTA and will study safety and efficacy of ISMN and/or cilostazol after lacunar stroke. 2. CVD-Cog, if funded by Alzheimer’s Society, will study ISMN and/or cilostazol in non-lacunar ischaemic stroke with radiological cSVD. 3. SVD-Cog will be submitted for funding and will study ISMN and/or cilostazol in patients attending a memory-dementia clinic who have radiological cSVD. Hence, ISMN would now be inappropriate to study in a platform
***Unranked***			
Atorvastatin (hydroxymethylglutaryl-CoA reductase-inhibitor). Alternative: rosuvastatin.	OR 1.44 (1.20, 1.72), N=923. Cost £1.32 pm. Score 16.4. Unranked since “would need more than just a statin”.	Licensed for primary and secondary prevention after vascular disease, including ischaemic stroke (80 mg od). Reduced stroke recurrence (N=4731). ^66^ No effect on impaired cerebrovascular reactivity or endothelial function in cSVD (N=94). ^67^ Improved digit symbol substitution test and reduced dementia. ^68^	Widely used after stroke but dementia/geriatric patients could be randomised.
Donepezil (acetyl cholinesterase-inhibitor, AChE-i). Alternatives: galantamine, rivastigmine	OR 1.52 (1.36-1.72), N=2354. Cost £1.22 pm. Score 16.3. Unranked since “no convincing mechanism or efficacy data”.	Licensed for early/medium AD (5 mg od). Reduced cognitive impairment in schizophrenia. ^69^ Meta-analyses compatible with improved cognition in VaD. ^41, 43^	All AChE-I VaD trials done only out to 6 months; no 18-month data. Metaanalysis influenced by a neutral CADASIL trial ^70^ although unclear how relevant monogenic cSVD treatments might be to sporadic cSVD? Cannot be trialled in mixed dementia since many will be on an AChE-I. Unclear whether AChE-Is have any neuroprotective role. ^44^
Lithium (glycogen synthase kinase 3-inhibitor, GSK3-i). No alternatives.	OR 2.52 (95% CI 1.63-3.90), N=289. Cost £7.50 pm. Score 15.7. Unranked “in view of monitoring requirements”.	Licensed for treatment and prophylaxis of mania, bipolar disorder and recurrent depression (1.0-1.5 gm od). No cSVD data. Adherence 85.6% at ? months. ^71^	Need to manage dose by plasma levels so multiple visits so adding complexity/expense.
Minocycline (tetracycline antibiotic). Alternative: doxycycline.	OR 1.66 (95% CI 1.22-2.30), N=547. Cost £13.02 pm. Score 16.2. Unranked because “unconvinced by earlier efficacy trials”.	Licensed antibiotic. Reduced cognitive impairment in schizophrenia. ^69^ Candidate treatment for AD. ^21, 22^	Adherence unreported for long-term therapy. No effect on cognition in AD (Nb avoid 200mg bd). ^45^ SLE (risk 2.6-8.5 fold) and liver dysfunction (risk 2.1 fold) increased if given for >1 year. ^72^
Remote ischaemic conditioning (RIC). No alternatives.	OR 2.76 (1.92-4.04), N=228. Cost unclear but likely to be significant. Score 24.9. Unranked since “not ready for large scale long-term use”.	Some devices have CE Mark. Reduces WMH/cSVD features. ^73– 76^ Improved cognition after ischaemic stroke. ^77^	Burden of self-administered long-term twice daily treatment. Poor adherence (46.5%) at 1 year. ^78^ Limited availability of MHRA-approved devices.
Semaglutide (glucagon-like peptide-1 receptor agonist, GLP-1ra). Alternative: liraglutide).	OR 2.04 (1.11-3.70), N=6480. Cost £78.48. Score 9.0. Unranked since trials funded for AD.	Licensed for type-2 diabetes. (Other GPL1ra are licensed for adjunct in weight loss). GLP-1ra reduce dementia in type-2 diabetes. ^79^ Cognition meta-analysis for any GLP1: OR 1.82 (0.95-3.50), N=7732. ^80^ No cSVD data. Adherence 69.7% at 12 months. ^81^	Too expensive so trial would need commercial support. Poor adherence rates.
Vitamin B6 & B9 (folic acid) & B12.	OR 0.95 (95% CI 0.87-1.04), N=6064. Cost unclear since VITATOPS/VISP drug doses are not available in UK. Score 14.7. Unranked since “large neutral trials already”.	Licensed for vitamin deficiencies, e.g. B12 deficiency. Periventricular white matter lucencies are related to low B12 levels in small vessel disease stroke. ^82^ Adherence 89.3% at 12 months. Positive mendelian randomisation study for stroke. ^53^	Neutral studies on recurrence after stroke (VISP, ^83^ VITATOPS ^84^). Neutral studies on cognition after stroke (VISP, ^83^ VITATOPS ^85^) and meta-analysis. Not available at the VITATOPS/VISP doses (over the counter doses are much lower). Would require 3 tablets

AD: Alzheimer’s disease; bd: twice daily; CI: confidence intervals; N: number; od: once daily; OR: odds ratio; pm: per month; SR: slow release.

### Independent review of 10 shortlisted drugs

The ISAG reviewed the summaries for 10 interventions. Three drugs were ranked: 1) metformin (biguanide, a metabolic modulator), 2) tadalafil (phosphodiesterase5-inhibitor causing vasodilation), and 3) isosorbide mononitrate (nitric oxide donor causing vasodilation). The other seven interventions were not considered to be relevant to study in a large trial for reasons that are given in Table 5, and so were not ranked: atorvastatin (hydroxymethylglutaryl-CoA reductase-inhibitor), donepezil (acetyl cholinesterase-inhibitor, AChE-i), lithium (glycogen synthase kinase 3-inhibitor, GSK3-i), minocycline (tetracycline antibiotic), remote ischaemic conditioning (RIC), semaglutide (glucagon-like peptide-1 receptor agonist, GLP-1ra) and vitamin B6/9/12.

Comparisons with recommendations for assessing interventions in other neurodegenerative conditions (AD, MND, MS) are given in Table 6. Some of the chosen interventions were not identified here for cSVD since they: are not in the BNF (e.g. fasudil, ibudilast, phenserine), ^29^ have significant side-effects (e.g. oxcarbazepine, perfenidone), were infrequently identified in preclinical screening (e.g. trazodone - only 4 mentions), lack safety information for long-term administration (e.g. anti-herpes drugs), are in widespread use ^35^ so would lead to a significant number of exclusions (angiotensin receptor antagonists, calcium channel blockers), have neutral effects in other neurodegenerative conditions (amiloride, fluoxetine, losartan, memantine, riluzole, trazodone), can be bought over the counter so potentially contaminating the control group (fatty acids, tadalafil, sildenafil, vitamins), or are already being tested (glucagon-like peptide-1 receptor agonists).

**Table 6.** Interventions prioritised for testing in neurological diseases following systematic assessment.

Drug class: examples	Alzheimer’s disease (AD)	Motor neurone disease (MND)	Multiple sclerosis (MS)	This review in cerebral small vessel disease (cSVD)
AChE-i: phenserine	Recommended for testing. ^22^			Not shortlisted here – not in BNF. ^29^
Anticonvulsant: Oxcarbazepine			Recommended for testing. ^23^	Not shortlisted here – significant side-effects.
Antidepressant: trazodone		Recommended for testing. ^24^ Neutral in MND-SMART (N=371). ^42^		Not shortlisted here – infrequently identified in preclinical screening (4 mentions), neutral in MND. ^42^
Anti-herpes: valaciclovir; valganciclovir	Recommended for testing. ^22^			Not shortlisted here – for short term use, not identified in pre-clinical search.
ARA: candesartan, losartan, telmisartan	Recommended for testing. ^21^ Losartan neutral (N=221). ^47^			Not shortlisted here – in widespread use; losartan neutral for AD. ^47^
Benzothiazoles: riluzole			Recommended for testing. ^23^ Neutral in MS-SMART (N=223). ^48^	Not shortlisted here – neutral in MS. ^48^
Biguanide: metformin			Recommended for testing. OCTOPUS is testing. ^33, 34^	Recommended here for testing.
CCB: amlodipine, nilvadipine	Recommended for testing. ^21^ Neutral (N=498). ^46^			Not shortlisted here – in widespread use; amlodipine (AFFECT) failed to recruit in cSVD; ^86^ nilvadipine not in BNF. ^29^
Fatty acids: linoleic acid, lipoic acid, omega-3 fatty acid			Recommended for testing. ^23^ OCTOPUS is testing R/S-alpha lipoic acid. ^33, 34^	Not shortlisted here – can be bought over the counter, omega-3 fatty acids.
NOD: ISMN				Recommended for testing here. ^12^ Positive, especially when co-administered with cilostazol. ^18^ To be tested in LACI-3.
GLP-1: liraglutide, semaglutide	Recommended for testing. ^21, 22^ ELAD is testing liraglutide. ^87^			Shortlisted but not ranked – already being tested, expensive and poor adherence.
NMDA-ra: memantine		Recommended for testing. ^24^ Neutral in MND-SMART (N=369). ^42^		Not shortlisted here – neutral in MND. ^42^
Pyridone immune-suppressive: perfenidone			Recommended for testing. ^23^	Not shortlisted here – severe side effects requiring specialist supervision. ^29^
PDE3-i: cilostazol				Recommended for testing. ^12^ Neutral in cSVD/positive if given with ISMN. ^18^
PDE4-i: ibudilast			Recommended for testing. ^23^	Not shortlisted here – not in BNF. ^29^
PDE5-i: tadalafil				Recommended here for testing.
Potassium-sparing diuretic: amiloride			Recommended for testing. ^23^ Neutral in MS-SMART (N=223). ^48^	Not shortlisted here – neutral in MS. ^48^
ROCK-i: fasudil	Recommended for testing. ^22^			Not shortlisted here – not in BNF. ^29^
SSRI: citalopram, fluoxetine, fluvoxamine, sertraline			Recommended for testing. ^23^ Fluoxetine neutral in MS-SMART (N=223). ^48^	Not shortlisted here – fluoxetine neutral in MS. ^48^
Tetracycline antibiotic: minocycline	Recommended for testing. ^21^ Neutral (N=544). ^45^			Shortlisted but not ranked – neutral for AD. ^45^

ARA: angiotensin receptor antagonist; CCB: calcium channel blocker; GLP-1: glucagon-like peptide-1 agonist; NMDA-ra: N-methyl-D-aspartate-receptor antagonist; NOD: nitric oxide donor; PDE5-i: phosphodiesterase5-inhibitor; ROCK-i: Rho-associated protein kinase-inhibitor; SSRI: selective serotonin reuptake inhibitor.

## Discussion

### Summary of evidence

Following screening of pre-clinical and clinical studies, we identified more than 1700 interventions that might be candidate treatments for cSVD. These were filtered down to ten by removing duplicates and interventions that were not possible or practical to assess in a phase-3 platform trial environment or that showed inadequate evidence of efficacy. The ten interventions comprised nine drugs (atorvastatin, donepezil, ISMN, lithium, metformin, minocycline, semaglutide and combined vitamin B6/9/12) and one device (remote ischaemic conditioning) ( Table 5). These were then ranked by an ISAG with three prioritised: a metabolic modulator (metformin) and two vasoactive agents (tadalafil, ISMN), all of which have some evidence that they may improve cognition in other conditions although none are licensed for this purpose.

The first ranked drug, metformin, a biguanide, is licensed for the treatment of type-2 diabetes mellitus (BNF ^29^) and guidelines recommend it for first-line therapy for this indication. It is also licensed for the prevention of diabetes in people with pre-diabetes. The meta-analysis showed that both the odds ratio (2.22) and lower 95% CI (1.66) were well above the target platform odds ratio of 1.40. Metformin has been identified for assessment in other neurological diseases and is being tested in the OCTOPUS platform trial in multiple sclerosis ( Table 6). ^33, 34^ However, a systematic review done for a guideline did not identify evidence supporting the use of antidiabetic drugs for treating cSVD although this recommendation was not specific to metformin. ^36^

The second ranked drug, tadalafil, is a phosphodiesterase5 inhibitor (PDE5-i) that maintains cyclic guanosine monophosphate levels, the second messenger for nitric oxide. It is licensed for the treatment of erectile dysfunction, benign prostatic hypertrophy and pulmonary hypertension (BNF ^29^) and has recently become available over the counter. The meta-analysis showed that both the odds ratio (7.91) and lower 95% CI (3.76) for PDE5-i were well above the target platform odds ratio of 1.40. However, the meta-analysis was based on a small dataset of 110 participants, mostly from before-after studies. Nevertheless, the positive findings for the third ranked drug, ISMN, a nitric oxide donor (NOD), in the LACI-2 trial ^18^ are supportive in view of the related mechanism of action for NOD and PDE5-i.

ISMN is licensed for the prophylaxis of angina and as an adjunct in congestive heart failure (BNF ^29^). Of the three drugs, ISMN is the only one to have been tested in cSVD, specifically in patients with previous lacunar infarction. The LACI-1 and LACI-2 trials showed that ISMN was feasible to take (given with or without cilostazol) for up to a year, was safe and improved cognition and functional outcome and reduced recurrent stroke, especially when given with cilostazol. ^18, 37– 39^ Recently, NIHR HTA funded the LACI-3 trial which will test the safety and efficacy of ISMN and cilostazol in patients with previous lacunar stroke in a 2x2 partial factorial design. Hence, it would now be inappropriate to test either of these drugs in a UK platform.

Practically, the only other short-listed intervention ( Table 5) that could readily be tested in a phase-3 platform for cSVD is one of the licensed oral AD drugs, as identified for VCI. ^40^ Eight short-term small-to-medium sized trials of AChE-i have been performed in VaD and they showed efficacy with an odds ratio (1.52) which exceeds the platform target odds ratio of 1.4; ^41^ the lower 95% CI boundary (1.36) just misses this. Donepezil at 10 mg daily would probably be the drug of choice since it appears to be more efficacious than when given at 5 mg daily ( Table 4). Memantine is also a possibility although it was neutral in MND-SMART. ^42^ A recent network metanalysis provides mixed results as to whether AChE-I improve cognition in VCI/VaD. ^43^ However, it remains unclear whether AChE-i and memantine only modulate symptoms in AD, also have some disease modifying/neuroprotective properties or even address AD pathology which may often be present in VCI/VaD. ^44^ In this respect, cSVD would be an interesting target for testing whether AChE-Is do have disease-modifying effects in view of the longer natural history for cognitive decline in cSVD.

The present study extended the methodology used previously for identifying candidate treatments for cSVD ^12^ by adding preclinical and clinical screening, mini-meta-analyses to assess whether an intervention might affect cognition in other disease areas and a scoring system. These approaches are broadly similar but not identical to those used for AD, MND and MS. ^21– 24^ Some relevant pathways and hence licenced drugs may have been missed due to restrictions on search terms – a future search should include other relevant pathways in other neurological diseases even if these have not yet been tested in animal models of cSVD. In retrospect, adding OR>1.40 or even the 95% lower CI >1.40 as a multiplier in scoring would have excluded short-listed interventions with little likelihood of success such as vitamin B12 ( Table 5).

Structured systematic approaches to identifying candidate treatments are not a guarantee that any identified and highly scored intervention will definitely work. For example, five classes of compounds were identified for repurposing as treatments of AD ^21^ and yet trials of minocycline, ^45^ nilvadipine ^46^ and losartan ^47^ were all neutral on clinical outcomes. ^22^ Similarly, of seven drugs identified for testing in MS, ^23^ amiloride, fluoxetine and riluzole were assessed at phase-2b and found to have no effect on MRI brain volume change between baseline and 96 weeks. ^48^ Recently, memantine and trazodone were found to be ineffective for MND. ^42^ Interestingly, all of these trials were much smaller than the planned UK STEP platform trial in cSVD and so underpowered for finding small but potentially useful clinical benefits at the population level providing the intervention is cost-effective. Further, some trials for neurogenerative conditions have relied on intermediate imaging endpoints which may be insensitive to changes in clinical endpoints; the incongruent relationship between neuroimaging intermediate outcomes at phase-2 and clinical functional outcomes at phase-3 has been highlighted for interventions such as citicoline for acute ischaemic stroke and blood pressure lowering for acute intracerebral haemorrhage. The framework for clinical trials in cSVD (FINESSE) recommends that clinical outcomes should be used as the main endpoint in cSVD trials although brain imaging changes may also be collected. ^49^ In this respect, LACI-2 showed that whilst clinical outcomes were positive, imaging outcomes were mostly neutral, suffered from larger data losses and come with increased costs and the need for participants to visit hospitals. ^50^

### Strengths and limitations

The main strength of this study is the triangulation of information from preclinical and clinical cSVD and VCI studies in the search for candidate treatments for cSVD, mini meta-analyses of potentially interesting interventions with scoring and then independent prioritisation and ranking. That only six of the ten shortlisted interventions were identified in clinical searches of completed, ongoing or planned trials shows the importance of also assessing the preclinical literature. Taken together, our approach offers a robust, efficient and reproducible approach to drug repurposing selection that could be used for other indications or target diseases.

However, there are also several limitations to the study. First, the intention was to identify potential interventions that could be tested at phase-3 by repurposing existing and inexpensive UK-licensed drugs. This meant we excluded drugs that are not available in the UK, could not logistically be administered long-term, were not ready for phase-3 trial assessment or were too expensive for widespread use. Hence, there will be novel interventions that could be tested at phase-2 and others that are available for repurposing outside of the UK. We excluded members of 62 classes of drugs that are not available in the UK; examples of drugs with potential relevance to cSVD include azelnidipine (calcium channel antagonist), beraprost (prostacyclin analogue), butylphthalide (neuroprotection and vasodilation), choline alphoscerate (neurotransmitter precursor), equol (oestrogen mimic), fasudil (rho-kinase inhibitor), ginkgo biloba (uncertain mechanisms), nerve growth factor and oxiracetam (nootrope), These are available in various parts of the world including low-middle income countries and some are in trial for cSVD ( Table 3). Equally, there will be drugs that are available in the UK but not in some other countries (such as isosorbide mononitrate).

Second, the review was performed rapidly to identify interventions that could be tested in a platform trial in response to a platform trial funding call. Although the time between call and grant submission was four months, the intervention identification phase only lasted two months since the platform design and its costing necessarily needed to know what interventions would be assessed initially. Further, electronic searches were limited to English language. As a result, it is likely that we have missed some studies and interventions although many were found as duplicates in searches. We are now extending the process to perform a deeper dive into phosphodiesterase inhibitors and acetylcholinesterase inhibitors/memantine and assess their effects on cognition in non-cSVD indications.

Third, the pre-clinical search focussed on models of relevance to cSVD and it is possible that this approach is too restrictive. Fourth, although the scoring system was based on one used previously, ^12^ it was expanded for use here without prior validation although it appears to have face validity. Fifth, we did not, in the main, account for dose in the meta-analyses. For the platform, we planned to initiate drugs at a low-medium dose and then escalate to near maximal, as we did in LACI-1/2 ^18, 38^ and will do in LACI-3. ^51^ Sixth, we did not seek evidence that interventions would alter mechanistic efficacy biomarkers. Seventh, the conversion of standardised mean difference to odds ratio for use in the mini-meta-analyses may have introduced bias or imprecision in effect estimates. And last, there are additional approaches that may be useful in identifying candidate drugs that we did not incorporate, for example Mendelian randomisation assessments ^52– 55^ and medication-wide association studies. ^56^

## Conclusions

In summary, we performed a rapid structured systematic search for interventions that might be relevant to treating or preventing cSVD. We identified interventions that could be repurposed and three were ranked by an ISAG; each of metformin, tadalafil and ISMN could readily be tested at phase-3 in the UK and be used routinely in clinical practice if found to be effective. Although our UK STEP grant application to test these in a platform environment was unsuccessful, we will be assessing ISMN as well as cilostazol in the recently funded LACI-3 trial.

### Ethics and consent

Ethics and consent were not required.

## Author contributions

• Philip M Bath: Conceptualisation, Data curation, Formal analysis, Investigation, Methodology, Project administration, Writing – original draft preparation
• Elizabeth P M Phan: Data curation, Formal analysis, Writing – review & editing
• Gwynneth Clay: Conceptualisation, Supervision, Writing – review & editing
• Jesse Dawson: Supervision, Writing – review & editing
• Paresh Malhotra: Supervision, Writing – review & editing
• Rob Howard: Supervision, Writing – review & editing
• Suvankar Pal: Supervision, Writing – review & editing
• Joanna M Wardlaw: Conceptualisation, Validation, Writing – review & editing
• Terry Quinn: Conceptualisation, Methodology, Supervision, Writing – review & editing
• Malcolm MacLeod: Conceptualisation, Data curation, Formal analysis, Investigation, Methodology, Resources, Writing – review & editing

## Units, symbols and mathematical scripts

There are no special units, symbols or mathematical scripts.

## Data Availability

Scoring of interventions for cerebral small vessel disease. http://doi.org/10.17639/nott.7493. ^57^ Attribution 4.0 International Deed license CC-BY 4.0 This project contains the following underlying data:
• Data file 1, Excel sheet giving scoring calculations: Scoring spreadsheet • Data file 2, Data dictionary for spreadsheet: Data Dictionary • Data file 3, PRISMA ScR checklist: PRISMA-ScR Checklist The report follows the checklist for the PRISMA extension for scoping reviews (PRISMA-ScR) guideline ^58^ adapted to account for the structure of this project and its multiple work packages. http://doi.org/10.17639/nott.7493. ^57^ Attribution 4.0 International Deed license CC-BY 4.0
