# Supplementary material for: Developing treatments for cerebral small vessel disease: a scoping review of licensed interventions for potential repurposing
Source: F1000Res. 2025 Sep 10;13:1546. Originally published 2024 Dec 20. [Version 2] doi: 10.12688/f1000research.157890.2 (PMC12335727; doi:10.12688/f1000research.157890.2)
Supplement: Peer review history

# Reviewer report: Reviewer response for version 1

**DOI:** 10.5256/f1000research.173409.r392606

**Published:** 2025-08-09

**Authors:** Maria Clara Zanon Zotin

**Affiliations:** 1 Universidade de Sao Paulo, São Paulo, State of São Paulo, Brazil

**This is a rapid multi-stage scoping review and mini-meta-analysis aimed at identifying potential interventions for preventing cognitive decline in patients with cSVD which could be tested in a phase-3 platform trial. Pre-clinical and clinical studies were screened. Potential interventions were filtered down to 10 by removing duplicates and those interventions deemed ineffective (including non-cost-effective ones) or unavailable. The shortlisted candidate interventions for cSVD were assessed in mini-meta-analyses, and phosphodiesterase5-inhibitors showed higher OR. These ten shortlisted interventions were further narrowed down to three by an independent scientific advisory committee that prioritised metformin, tadalafil, and ISMN.**

**This is a well-structured and well-written straight-forward and practical scoping review of experts in the field of CSVD and vascular dementia, aimed at guiding future research and fostering purposeful clinical trials. This review contributes significantly to the field by focusing on repurposing existing drugs, which may lead to faster and more cost-effective future trials. I have only a minor questions/comment:**

 The authors clearly state that the study aims to identify promising drugs suitable for phase-3 testing in the UK and acknowledge this location-specificity as a limitation, given that drugs available elsewhere may be excluded. Could the authors comment on how restricting the analysis to UK-authorized drugs and excluding “impossible or impractical interventions” might affect the generalizability of the findings to other countries, particularly low- and middle-income settings where CSVD burden is highest? From my perspective, these restrictions may not diminish, and could potentially enhance, the applicability of the results to resource-limited contexts with constrained research funding and healthcare infrastructure. The authors’ insights on this would be valuable.

Are the rationale for, and objectives of, the Systematic Review clearly stated?

Yes

Is the statistical analysis and its interpretation appropriate?

Yes

If this is a Living Systematic Review, is the ‘living’ method appropriate and is the search schedule clearly defined and justified? (‘Living Systematic Review’ or a variation of this term should be included in the title.)

Yes

Are sufficient details of the methods and analysis provided to allow replication by others?

Yes

Are the conclusions drawn adequately supported by the results presented in the review?

Yes

Reviewer Expertise:

Neuroradiology. Vascular Neurology. Cerebral Small Vessel Diseases.

I confirm that I have read this submission and believe that I have an appropriate level of expertise to confirm that it is of an acceptable scientific standard.

# Reviewer report: Reviewer response for version 1

**DOI:** 10.5256/f1000research.173409.r392611

**Published:** 2025-07-22

**Authors:** Jade L. Taylor

**Affiliations:** 1 University of California Davis, Davis, California, USA

The systematic review by Bath et al. presents a well-designed and thoughtfully executed synthesis of both clinical and preclinical trials investigating potential therapies for cerebral small vessel disease (cSVD). The authors employ a robust multi-scale approach, integrating preclinical screening, feasibility scoring, and evaluation by an independent scientific advisory group (ISAG), which collectively enhance the strength and credibility of the report. Practical considerations, such as cost and long-term tolerability of interventions, are sensibly incorporated, increasing the likelihood that identified candidate therapies will be viable in clinical practice.

 Importantly, the authors are transparent about the absence of a pre-registered protocol (e.g., PROSPERO), acknowledging the limitation this poses in terms of replicability. Given the current lack of effective disease-modifying treatments for cSVD, this review represents a timely and valuable contribution to the field.

**Areas for revision or clarification:**

1. Page 2, Paragraph 2 (Results section of the abstract): The phrase “those that were *not* available in the UK” should be corrected to “those that were available in the UK.”
2. ISAG Criteria: The criteria employed by the ISAG should be introduced earlier in the manuscript—preferably in the methods section—and clearly linked to Table 5 for clarity.
3. The inclusion of only English-language studies should be explicitly acknowledged as a limitation, as it may have resulted in the exclusion of relevant non-English literature.
4. The limitations of converting standardized mean differences from before-after studies to odds ratios for comparison purposes should be discussed, as this transformation may introduce bias or imprecision in effect estimates.

Are the rationale for, and objectives of, the Systematic Review clearly stated?

Yes

Is the statistical analysis and its interpretation appropriate?

I cannot comment. A qualified statistician is required.

If this is a Living Systematic Review, is the ‘living’ method appropriate and is the search schedule clearly defined and justified? (‘Living Systematic Review’ or a variation of this term should be included in the title.)

No

Are sufficient details of the methods and analysis provided to allow replication by others?

Yes

Are the conclusions drawn adequately supported by the results presented in the review?

Yes

Reviewer Expertise:

cerebral blood vessel physiology and pathophysiology

I confirm that I have read this submission and believe that I have an appropriate level of expertise to confirm that it is of an acceptable scientific standard.

# Response

**Published:** 2025-08-11

**Authors:** Philip Bath

**Affiliations:** Stroke Trials Unit, University of Nottingham, Nottingham, Notts, UK

We thank Dr Taylor for their helpful comments.

1. Page 2, Paragraph 2 (Results section of the abstract): The phrase “those that were *not* available in the UK” should be corrected to “those that were available in the UK.”

**Thank you – we have corrected this in Abstract/Results..**

**CHANGE**

1. ISAG Criteria: The criteria employed by the ISAG should be introduced earlier in the manuscript—preferably in the methods section—and clearly linked to Table 5 for clarity.

**Thank you – we have expanded on the criteria assessed by ISAG in the Methods/Independent review section with a link to Table 5.**

**We have also corrected the use of Independent Scientific Advisory Committee (ISAC) by changing to … Group (ISAG) in the Header of Table 5 and text (sections Overview, Table 3 and Independent review…) for consistency with the original NIHR research call.**

**CHANGE**

1. The inclusion of only English-language studies should be explicitly acknowledged as a limitation, as it may have resulted in the exclusion of relevant non-English literature.

**Thank you – we have added this point to the Discussion/limitations section (added to limitation 2.**

**CHANGE**

1. The limitations of converting standardized mean differences from before-after studies to odds ratios for comparison purposes should be discussed, as this transformation may introduce bias or imprecision in effect estimates.

**Thank you – we have added this point to the Discussion/limitations section (new limitation 7).**

**CHANGE**
